# Clinical characteristics of a large cohort of patients with positive culture of Fusobacterium necrophorum

**DOI:** 10.3205/id000038

**Published:** 2018-03-07

**Authors:** Romana Klasinc, Kostiantyn Lupyr, Iris Zeller, Helga Paula, Athanasios Makristathis, Felix Tuchmann, Thomas Wrba, Ojan Assadian, Elisabeth Presterl

**Affiliations:** 1 Department of Infection Control & Hospital Epidemiology, Medical University of Vienna, Austria; 2 Institute for Hygiene and Applied Immunology, Center for Pathophysiology, Infectiology and Immunology, Medical University of Vienna, Austria; 3 Division of Clinical Microbiology, Department of Laboratory Medicine, Medical University of Vienna, Austria; 4 Department of Dermatology, Medical University of Vienna, Austria; 5 Medical University of Vienna, IT4Science, IT-Systems & Communications, Vienna, Austria

**Keywords:** Fusobacterium necrophorum, Lemierre’s disease, anaerobe, sepsis, thrombosis

## Abstract

**Background:** *Fusobacterium necrophorum* is a rare pathogen, mostly affecting young adults, causing infections of the head and neck, typically described as the Lemierre’s syndrome. Today this symptom complex has become increasingly rare and has almost turned to a ‘forgotten disease’.

**Methods:** We performed a retrospective, descriptive study to identify the clinical features of patients with positive culture of *F. necrophorum*. Additionally, the antibiotic susceptibility profile of the pathogens was analysed.

**Results:** During a period of 22 years 36 patients with at least one isolate of *F. necrophorum* were identified. Mostly tonsillar and peritonsillar abscesses were found, 10 patients were identified with bacteraemia, but only 4 patients presented with symptoms like sore throat, fever and swollen cervical lymph nodes, which may suggest Lemierre’s. Most of the isolates (33/35) showed sensitivity to all tested antibiotics.

**Conclusion:** Appropriate techniques are needed to detect *F. necrophorum*, especially from throat swabs, in the microbiological laboratory. Current clinical and microbiological practice may lead to under-diagnosis of infections caused by *F. necrophorum*. Further research is needed to define the colonization rate and to optimize methods for detection as well as identification of virulence.

## Background

Infections caused by *Fusobacterium necrophorum* may result in human necrobacillosis characterized as sore throat, bacteremia, multiple abscesses, jugular vein thrombosis and metastatic septic embolization [1]. This symptom complex was described first by André Lemierre in 1936 in a case series of 20 patients [2] where throat infections were followed by anaerobic septicemia caused by “*Bacillus funduliformis*” (today: *F. necrophorum*). All but 2 of those initially described 20 patients died. In the 1960s and 1970s, possibly due to development of antibiotic therapy, Lemierre’s disease became increasingly rare and today has almost turned to a ‘forgotten disease’ [3]. 

Fusobacteria are Gram-negative, non-spore-forming anaerobic rods [4]. The taxonomy of the genus *Fusobacterium* includes 15 species, of which *F. nucleatum* and *F. necrophorum* are the species most frequently found in conjunction with clinical disease. *F. necrophorum* is a common inhabitant of the human digestive system and is found in the oral cavity, the upper respiratory tract as well as in the vaginal mucosa [5], [6]. *F. necrophorum* is divided in two subspecies: *F. necrophorum* ssp. *necrophorum*, which mostly causes infections in animals, and *F. necrophorum* ssp. *fundiliforme* causing infections in animals as well as humans [7].

*F. necrophorum* affects mostly young, healthy individuals [8], causing infections of the head like tonsillitis, peritonsillar abscess, post-anginal cervical lyphadenitis, otitis media in children, and sinusitis in adults [2], [9]. Furthermore, the pathogen may cause a persistent sore throat syndrome (PSTS) presenting with high fever, general malaise, lymphadenitis, tonsillar lesions and dysphagia during the acute phase [10]. 

A number of case series with *F. necrophorum* infections have been described in previous studies. Pett et al. [11] reported on a series of *Fusobacterium* spp. infection in 18 patients. This study, however, included also *F. varium* and *F. nucleatum*. Another study investigating Lemierre’s disease observed selectively *F. necrophorum* infection, which, however, included only 3 patients [12]. Here, we report on 36 patients with solely *F. necrophorum* infection, which may represent one of the largest case series on patients with *F. necrophorum* infection in the literature so far.

## Methods

In order to identify all microbiological samples positive for *F. necrophorum* at the Vienna University Hospital, a 1,922-bed tertiary-care university teaching hospital, a retrospective search of all electronic records, obtained in the time period between June 1995 and January 2017, was performed. The clinical information and antimicrobial susceptibility test results were obtained from the Research, Documentation & Analysis (RDA) IT-platform and the hospital information system (HIS) of the Vienna General Hospital. The RDA platform is an IT system for the integrated support of medical research at the Medical University of Vienna (MUW), which provides the scientific research data in a highly structured form in a central database, which can be used for further specific queries and data download.

Samples were cultured following the standard operating procedures implemented at the Division of Clinical Microbiology. Samples consisted of blood cultures, swabs, and aspirates. Blood cultures were incubated for up to 7 days at 36.5–37°C in the BacT/ALERT 3D system (BioMérieux, Marcy l’Etoile, France). Gram stains and subcultures were performed from positive blood cultures. Identification to the species level was done using the Vitek II system (BioMérieux) and after 2010 by the MALDI Biotyper instrument (Bruker Daltonics GmbH, Bremen, Germany). For the detection of Gram-negative, anaerobic bacteria in aspirates and swabs obtained from abscess areas, those were cultured under anaerobic conditions at 35–37°C on Schaedler Kanamycin-Vancomycin plates (Becton Dickinson) for 48 h. From throat swabs cultures aiming at the detection of true anaerobes – as described above – were only conducted if the clinical information enclosed indicated the possible involvement of Fusobacterium (i.e. chronic tonsillitis). For antimicrobial susceptibility testing the minimum inhibitory concentration (MIC) was determined using the E-test (BioMerieux, Marcy l’Etoile, France and AB Biodisk, Solna, Sweden) following the manufacturer’s instructions. Since recurrent bacteraemia with an identical strain may have occurred and would have biased the cumulative susceptibility profile, only the first isolate per patient during the study period was included into this analysis.

### Statistical analysis

Data and parameters were extracted from the RDA platform as *.csv file and collected in line-lists using MS Excel (Microsoft Excel 2010, version 14.0.4763.1150; Microsoft, Richmond, USA), which was also used to perform the descriptive statistics. Categorical data were expressed by percentage, continuous variables were expressed as mean and standard deviation (± SD) if normally distributed.

## Results

During 1995 until 2017, 36 patients with at least one isolate of *F. necrophorum* were identified. Patient and specimen details are summarized in Table 1 (Tab. 1). The patients’ age ranged from 3 to 76 years (mean age ± sd: 36 ± 4 years). Among these patients, 14 were female and 22 were male. Eight patients presented with infections in the head and neck region. Sore throat, fever and swollen cervical lymph nodes, possible indicators of Lemierre’s disease, were found in 4 patients. Of these, one patient (female, 19 years of age) presented with an acute lymphadenitis on the face, head and neck. Furthermore, a patient (male, 28 years of age) yielded *F. necrophorum* in blood culture and was hospitalized due to symptoms of a phlegmon of the sternoclavicular joint. However, the examined medical records did not contain information if he also presented with tonsillitis. Another patient (male, 14 years of age) had a sub-pectoral abscess with clinical signs of inflammation in this anatomic region and suspected infestation of the lymph nodes. The fourth patient (male, 20 years of age) with possible Lemierre’s disease presented with painful neck, cervical lymphadenitis, pneumonia and sepsis. One third of the patients (n=12) presented solely with a *F. necrophorum* infection, while all other patients showed multi-microbial flora. Most patients with mono-microbial infections belonged to the age group of 16–30 and presented with abscesses in different anatomic regions (tonsils, sub-mental, fossa canina, tubo-ovarial, pectoral, lungs, submandibular and pterygomandibular). The clinical characteristics of all cases are summarized in Table 1 (Tab. 1).

Overall, most isolates were sensitive to all tested antibiotics. Only 2 of 35 tested isolates showed resistance, one isolate against penicillin G and the second against metronidazole. The results of *in-vitro* susceptibility testing of isolates are summarised in Table 2 (Tab. 2).

## Discussion

Although there are several reports highlighting that *F. necrophorum* is an important cause of bacterial pharyngitis with prevalence as high as group A Streptococci in adolescents and young adults [13], [14], our investigation identified only 36 patients with positive *F. necrophorum* culture during a period of over 20 years. Only 8 of these 36 patients yielded *F. necrophorum* from clinical samples obtained from the upper respiratory tract. In our case series not a single case of jugular vein thrombosis was described, also none of the cases presented as typical Lemierre’s syndrome. However, 4 patients presented with neck swelling possibly suggestive for Lemierre’s syndrome, but the medical records of these patients did not contain information if Lemierre’s syndrome was suspected clinically.

Overall, the most common clinical features in younger patients were tonsillitis, peritonsillar abscess, post-angina cervical lymphadenitis, or otitis media. In older individuals *F. necrophorum* mostly was yielded in poly-microbial cultures from gingival and periodontal affections, but was also isolated from the abdominal cavity.

Interestingly, 22 of 36 of patients with *F. necrophorum* infection were male. This observation is in line with a previous case series published by Pett et al. [11] where 9 infections with *F. necrophorum* were reported, all in young, male patients. Patients with underlying diseases were more likely to be infected with *F. nucleatum* than *F. necrophorum*. This was demonstrated in a case series including 52 patients with *Fusobacterium* sp. bacteraemia, 23 of them infected with *F. necrophorum* [15]. Another case series, including 40 cases of *Fusobacterium* sp. bacteraemia (thereof 8 patients presenting with *F. necrophorum*), reported frequently nosocomial infections, occurring mostly in males with underlying diseases [16]. Afra et al. [8] estimated the overall annual incidence of bacteraemia due to *Fusobacterium* sp. at 0.55 cases per 100,000 populations and identified *F. necrophorum* in 18 cases out of 72 cases of *Fusobacterium* spp. bacteraemia. Other case series included a very small number of patients (3 to 9 cases), with little information to draw further conclusions [17], [18], [19], [20]. A similar incidence for *F. necrophorum* infection was reported in a prospective study reporting rates of 0.31–0.83 cases per 100,000 inhabitants per year [21], suggesting *F. necrophorum* being the second most common bacterial cause of pharyngo-tonsillitis [22]. In several studies isolation of *F. necrophorum* followed in 10–48% of cases of persistent, recurrent and chronic sore throats [1], [23], [24] and was isolated in 28% in patients subjected to tonsillectomy [25]. The prevalence of *F. necrophorum* in children was significantly higher among young adults aged 14 to 20 years (14%), than in patients younger than 14 years (2%) [26]. 

According to the “UK Standards for Microbiology Investigations B9/Investigation of throat related specimens” bacteriologic work-up of specimens for *F. necrophorum* is only recommended in the clinical cases of persistent sore throat or quinsy [27]. Based on our results it may be questioned if such microbiological processing algorithm represents an effective and reliable method to detect *F. necrophorum* in the upper respiratory tract, and hence early detection of Lemierre’s Syndrome.

Centor [14] highlighted that the risk for the Lemierre’s syndrome after *F. necrophorum* pharyngitis greatly exceeds the risk for acute rheumatic fever after group A beta-haemolytic streptococcal pharyngitis. Therefore the diagnostic paradigm of pharyngitis in adolescents and young adults should be expanded and improved detection methods should be considered. Another important aspect is the fact that *F. necrophorum* is able to aggregate platelets and causes thrombosis. In a study published by Forrester et al. [28] the ability of *F. necrophorum* (strains 3080 and 5018) to induce platelet aggregation was demonstrated. Interestingly, in this study aggregation was demonstrated only in biotype A (subsp. *necrophorum)* and could not be demonstrated in biotype B (subsp. *funduliforme)* or the AB biotype, indicating a strain-dependent virulence [28]. In literature subsp. *necrophorum* is described as more virulent, but the isolation mainly follows from infections in animals [29]. In this context, it is worth to highlight the importance of diagnostic methods, including PCR based methods, for reliable identification of *F. necrophorum* to subspecies level. Jensen et al. [30], [31] demonstrated in detailed investigations that all isolates except for 1 were identified as *F. necrophorum* subsp. *funduliforme*.

The determination of the species and subspecies together with their potential virulence, as well as the effect on blood platelets aggregation, should be considered in order to identify potential serious complications at an early stage. It might not be sufficient to solely detect *F. necrophorum* in clinical samples. The identification of the subspecies present and its potential to influence the kinetics of aggregation might be more important for the prevention of complications. 

Our case series is limited by the fact that only patients with positive microbiological identification of the pathogen were included, while patients with no microbiological investigation or false negative results were not noted even though they might have had clinical symptoms suggesting Lemierre’s syndrome. Therefore, the number of clinical cases diagnosed as Lemierre’s syndrome without microbiological identification remains unknown. Furthermore, the identification of *Fusobacterium* sp. did also not include the identification of the subspecies, since this was and is not routine microbiological procedure.

In conclusion further research is needed to optimize the identification of *F. necrophorum* from throat swabs, to discriminate between virulent species and to identify their role in causing complications or as colonizing organisms. In the group of adolescents and young adults with sore throat targeted investigation for the presence of *F. necrophorum* should be considered for routine diagnostics. In addition, due to the possible complication of thrombosis and abscess formation, imaging in all infections with *Fusobacterium* sp. should be considered.

## Notes

### Competing interests

The authors declare that they have no competing interests.

### Funding

This work was supported by internal funding.

## Figures and Tables

**Table 1:**
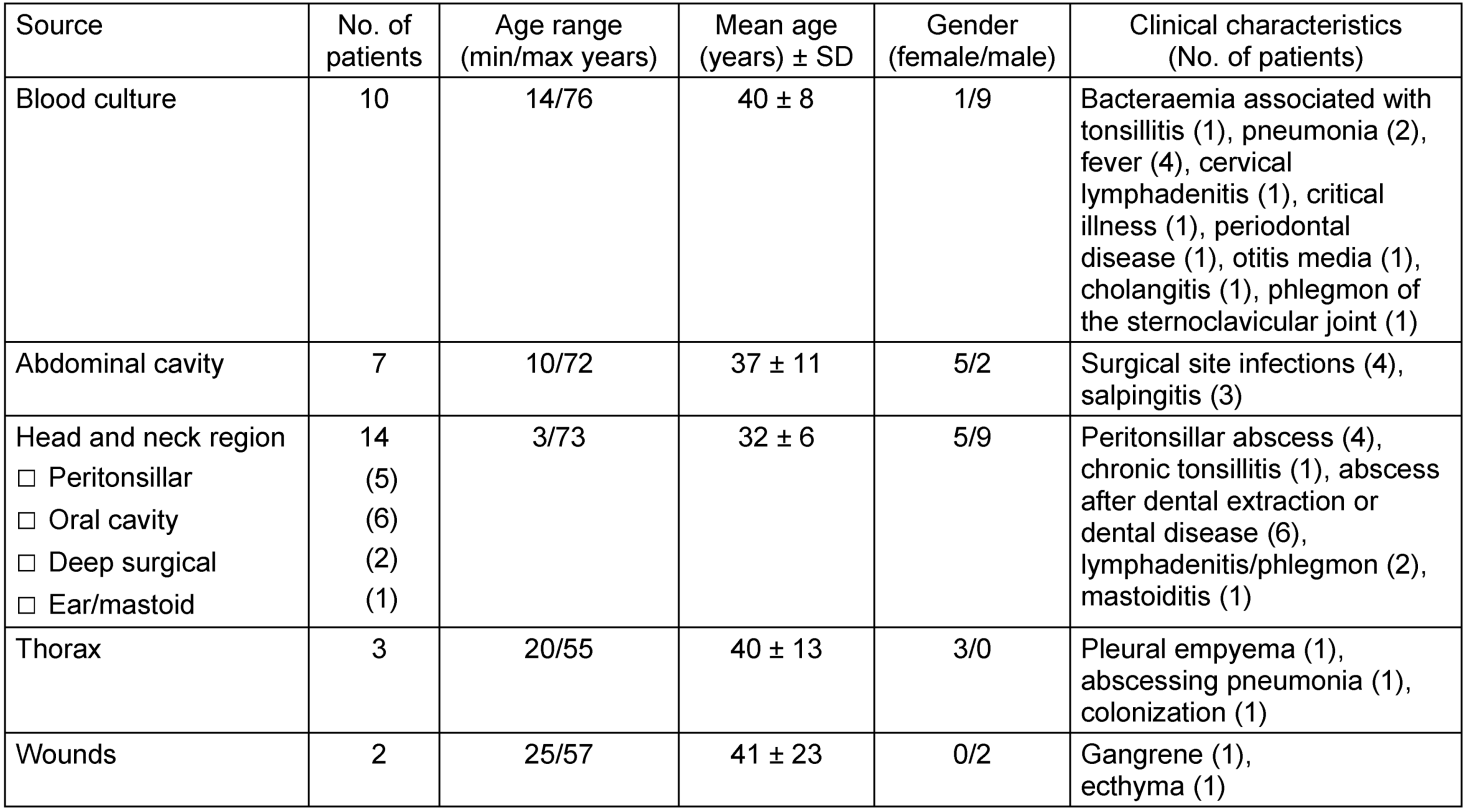
Samples with positive *F. necrophorum* culture categorized according to source and clinical characteristics

**Table 2:**
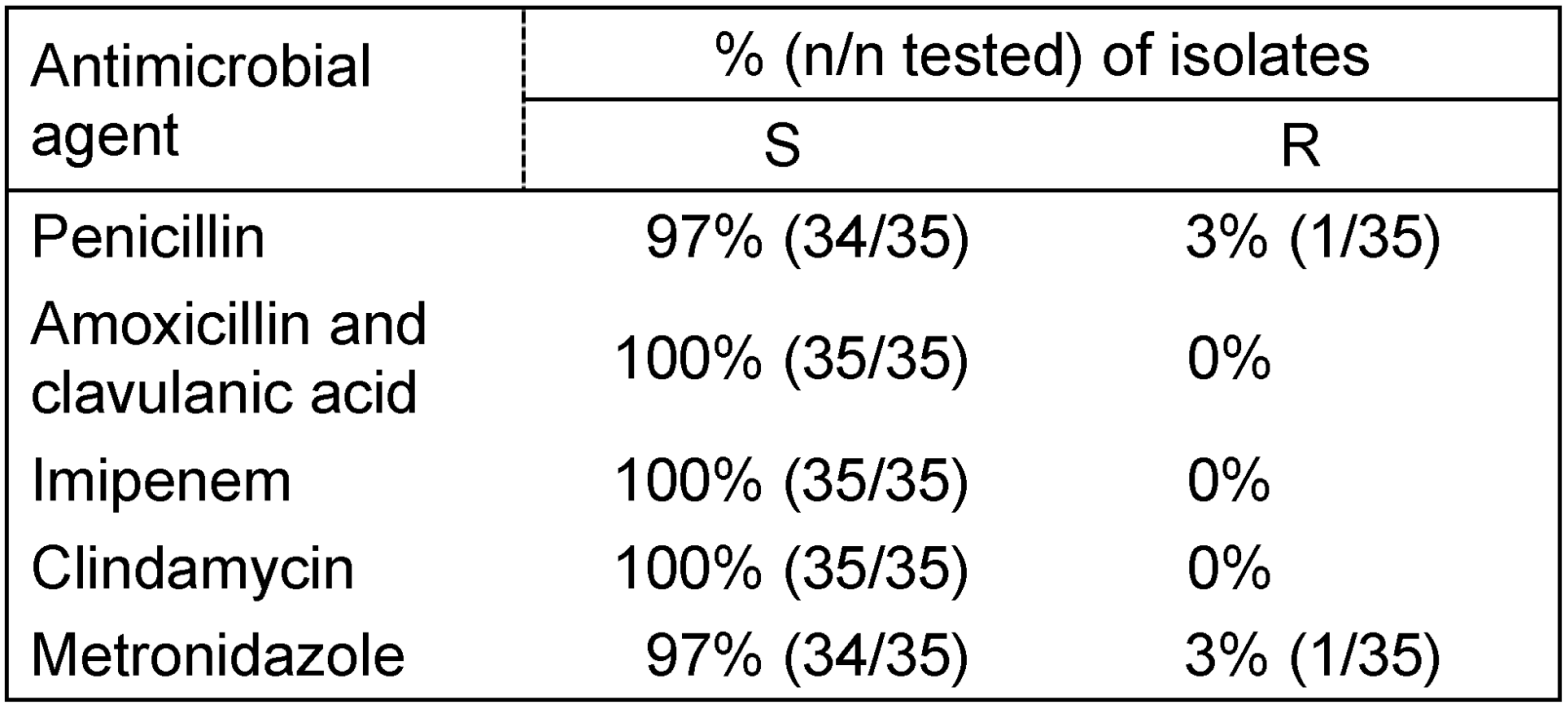
Antibiotic susceptibility profile
